# A mycotic aneurysm related to *Salmonella* Rissen infection: a case report

**DOI:** 10.1186/s12879-020-4819-0

**Published:** 2020-01-31

**Authors:** Jakub Nagrodzki, Katherine E. Sharrocks, Vanessa K. Wong, Andrew J. Carmichael

**Affiliations:** 1 School of Clinical Medicine, University of Cambridge, Cambridge, CB2 0SP UK; 2 Trinty College, University of Cambridge, Cambridge, CB2 1TQ UK; 3 Infectious Diseases Department, Addenbrooke’s Hospital, Cambridge, CB2 0QQ UK; 4 Microbiology Department, Addenbrooke’s Hospital, Cambridge, CB2 0QQ UK

**Keywords:** Mycotic aneurysm, *Salmonella* Rissen, Endovascular infection

## Abstract

**Background:**

*Salmonella* species commonly causes infection in humans and on occasion leads to serious complications, such as mycotic aneurysms. Here, we present the first case reported of a patient with a mycotic aneurysm likely secondary to *Salmonella* Rissen infection.

**Case presentation:**

The patient presented with 4 weeks of lower back pain, chills and a single episode of diarrhoea 2 months prior during a 14-day trip to Hong Kong and Taiwan. Magnetic resonance imaging revealed an aneurysmal left internal iliac artery with adjacent left iliacus rim-enhancing collection. A stool culture was positive for *Salmonella* Rissen ST 469 EBG 66 on whole genome sequencing. The patient underwent an emergency bifurcated graft of his internal iliac aneurysm and was successfully treated with appropriate antibiotics.

**Conclusions:**

This case highlights the importance of considering the diagnosis of a mycotic aneurysm in an unusual presentation of back pain with features of infection.

## Background

*Salmonella enterica subsp. enterica* is one of the leading causes of gastroenteritis and bacteraemia worldwide, including the European Union [1, 2]. Whilst non-typhoidal *Salmonellae* infection typically presents with gastroenteritis and fever and resolves without serious complications, certain serovars may cause systemic disease, especially in immunocompromised patients [1, 3]. One of the most serious complications of *Salmonella* is endovascular infection, the incidence of which is reported to be 25 to 35% of patients over 50 years old with bacteraemia [4]. Diagnosis, treatment and prognostic outcomes for patients with mycotic aneurysms related to *Salmonella* spp. are poor [5, 6].

The case presented here is the first case reported in literature of a patient with a mycotic aneurysm secondary to *Salmonella enterica subsp. enterica* serovar Rissen infection, successfully treated with surgery and antibiotics.

## Case presentation

A 69-year-old chef with a history of hypertension and a coronary artery bypass graft presented to hospital with several weeks of severe back pain, pain down his left lower limb and weight loss. During that time, he reported one episode of vomiting and chills, but no fever. Two months prior, he had spent 14 days in Hong Kong and Taiwan. He had a single episode of loose stool on this trip. On examination, the patient was apyrexial and haemodynamically stable with unremarkable cardiovascular, respiratory and abdominal examinations.

Blood tests revealed the following: haemoglobin 118 g/L, mean cell volume 73.3 fL, erythrocyte sedimentation rate 100 mm, C-reactive protein 87 mg/L, ferritin 1000 μg/L and a white cell count of 8.4 × 10^9^/L (normal differential), lactate 2.0 mmol/L, urea 5.9 mmol/L, creatinine 78.2 μmol/L, normal electrolytes and liver function tests.

Microbiological investigations included three sets of blood cultures and a urine culture, which were negative. Hepatitis B surface antigen, hepatitis C antibody, HIV antigen/antibody, and syphilis serology were negative. A stool culture was positive for *Salmonella* species sensitive to azithromycin (minimum inhibitory concentration (MIC) 6.0 mg/L), ciprofloxacin (0.008 mg/L), amoxicillin/clavulanate (3.0 mg/L), and resistant to sulfamethoxazole/trimethoprim (32 mg/L) amoxicillin (EUCAST disc diffusion diameter 7 mm), chloramphenicol (16 mm), and trimethoprim (7 mm). This isolate was identified as *Salmonella* Rissen ST 469 EBG 66 sensitive to ceftriaxone (MIC 0.125 mg/L) by the reference laboratory Public Health England, UK, using whole genome sequencing.

**Fig. 1:**
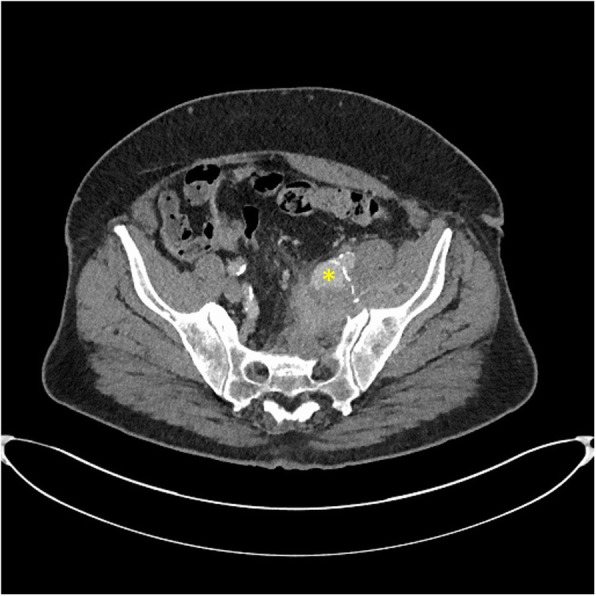
The patient’s contrast CT scan (pelvis) showing a left internal iliac artery mycotic aneurysm (*)

The patient had an unremarkable oesophago-gastro-duodenoscopy and colonoscopy. Computer tomography (CT) of the abdominal aorta with contrast and a magnetic resonance imaging of the pelvis and spine revealed a left pelvic sidewall mass (35 mm) centred on an aneurysmal left internal iliac artery (Fig. 1), causing its occlusion, with adjacent rim-enhancing collection within the left iliacus in keeping with an abscess and likely mycotic aneurysm, as well as an infra-renal abdominal aortic aneurysm measuring 51.8 mm. A CT-guided biopsy of the left iliacus muscle revealed mild chronic inflammation. Microbiological culture and 16S polymerase chain reaction from this sample were negative (primers and targets as described in literature [7].

A few days later the patient underwent a bifurcated graft of his internal iliac aneurysm, receiving 1000 mg flucloxacillin and 120 mg gentamicin intravenously at induction for vascular surgery prophylaxis (rather than specific *Salmonella* treatment). No collections were noted intraoperatively. He was initially treated with intravenous amoxicillin/clavulanate (1000 mg/200 mg) three times daily 11 days after admission with some clinical improvement. Oral ciprofloxacin 500 mg twice daily was added 19 days after his admission. After 3 weeks in hospital, he was discharged on oral ciprofloxacin 500 mg twice daily and amoxicillin/clavulanate (500 mg/125 mg) three times daily as treatment for a presumed *S.* Rissen mycotic aneurysm. Amoxicillin/clavulanate was continued to cover the possibility of poly-microbial infection, as the patient showed initial clinical response to this agent.

At his 6 months follow-up review in clinic the patient was well and was taking his antibiotic treatment without side effects. A CT scan at 11 months showed ongoing inflammatory changes at the site of the graft, which may represent ongoing infection, and a decision was made to rationalise his antibiotics to life-long azithromycin suppressive treatment. He has since submitted three culture-negative stool samples and has been allowed to return to work as a chef.

## Discussion and conclusions

The unusual nature of this case lies in the causative organism. As far as we can tell, there have been no cases of a mycotic aneurysm in literature where *S.* Rissen was isolated.

Cases of *Salmonella* mycotic aneurysms in literature are dominated by males above the age of 60, who suffer from hypertension, diabetes mellitus and atherosclerosis – similar co-morbidities to our patient [4, 5]. Atherosclerosis predisposes to endothelial invasion by *Salmonella* [8] and in this case was one of the most significant risk factors.

Most commonly, mycotic aneurysms present with fever (over 80%), chills, pain around the location of the aneurysm (back or abdominal pain) and sporadic diarrhoea, but on occasion prolonged back pain is the only presenting symptom [4, 5].

An important aspect of the case is the history of the patient’s foreign travel. In Europe, most *Salmonella* infections are caused by serovars Typhimurium and Enteritidis, although recently an increased number of cases caused by serovar Rissen in Thailand, Cambodia, Denmark, Italy and Spain was reported, predominantly resulting from pork ingestion [9–11]. Recently, high prevalence of *S.* Rissen in chicken and pork meat at retail markets in Eastern China – a region close to Taiwan and Hong Kong, where the patient presumably acquired the infection – was reported [12].

In the majority of cases, isolates of the causative organism are obtained both from blood and the aneurysm itself [4, 13]. It is, therefore, unusual that in this case blood cultures remained negative and the bacterium was only isolated from stool. Blood cultures, however, may have poor sensitivity and specificity in the diagnosis of mycotic aneurysms [14].

The main locations of aneurysms determined by imaging are abdominal or thoracic aorta and aortic arch, and only in the minority of cases the iliac artery and beyond [4, 5, 13]. Aneurysm-associated psoas abscesses are not unusual in *Salmonella* infection [5].

Surgical treatment focuses on endovascular aneurysm repair due to faster recovery than open repair [5, 15]. Here, open repair with grafting was preferred because of a lower rate of re-intervention. In terms of antibiotic therapy, *Salmonella* mycotic aneurysms are usually treated with quinolones or 3rd generation cephalosporins, and only rarely with β-lactam antibiotics, for at least 24 weeks post-operatively [5]. Limited data on antimicrobial susceptibility are available, but multidrug-resistant isolates of this serovar have already been reported [9].

The key take-away messages of this case are, firstly, the consideration of mycotic aneurysm in patients with cardiovascular risk factors presenting with back pain accompanied by chills or fever, and with a history of sporadic diarrhoea. Secondly, a diagnosis of a mycotic aneurysm should not be excluded based on negative blood cultures. Thirdly, mycotic aneurysms secondary to *Salmonella* infection can be successfully treated with open surgery and antibiotics, which may have to be life-long due to risk of a potentially fatal graft infection.

## Data Availability

Data sharing is not applicable to this article, as it includes personal medical information only accessible to healthcare professionals relevant to the care of this particular patient. No datasets were generated or analysed during this study.

## Abbreviations

CT: Computer tomography
MIC: Minimum inhibitory concentration

## References

[1] Bonardi S (2017). Salmonella in the pork production chain and its impact on human health in the European Union. Epidemiol Infect.

[2] Hendriksen RS, Vieira AR, Karlsmose S, Lo Fo Wong DMA, Jensen AB, Wegener HC (2011). Global monitoring of *Salmonella* serovar distribution from the World Health Organization Global Foodborne Infections Network Country Data Bank: results of quality assured laboratories from 2001 to 2007. Foodborne Pathog Dis.

[3] Pham OH, McSorley SJ (2015). Protective host immune responses to Salmonella infection. Future Microbiol.

[4] Wang J-H, Liu Y-CL, Yen M-Y, Wang J-H, Chen Y-S, Wann S-R (1996). Mycotic aneurysm due to non-typhi Salmonella: report of 16 cases. Clin Infect Dis.

[5] Guo Y, Bai Y, Yang C, Wang P, Gu L (2018). Mycotic aneurysm due to Salmonella species: clinical experiences and review of the literature. Braz J Med Biol Res.

[6] Andrews JR, Ryan ET (2015). Diagnostics for invasive Salmonella infections: current challenges and future directions. Vaccine.

[7] Harris KA, Hartley JC (2003). Development of broad-range 16S rDNA PCR for use in the routine diagnostic clinical microbiology service. J Med Microbiol.

[8] Laohapensang K, Rutherford RB, Arworn S (2010). Infected aneurysm. Ann Vasc Dis.

[9] Hendriksen RS, Bangtrakulnonth A, Pulsrikarn C, Pornreongwong S, Hasman H, Song SW (2008). Antimicrobial resistance and molecular epidemiology of *Salmonella* Rissen from animals, food products, and patients in Thailand and Denmark. Foodborne Pathog Dis.

[10] Boschi T, Aquilini D, Degl’Innocenti R, Aleo A, Romani C, Nicoletti P (2007). Cluster of cases of *Salmonella enterica* serotype rissen infection in a General Hospital, Italy. Zoonoses Public Health.

[11] Nadimpalli M, Fabre L, Yith V, Sem N, Gouali M, Delarocque-Astagneau E (2019). CTX-M-55-type ESBL-producing Salmonella enterica are emerging among retail meats in Phnom Penh, Cambodia. J Antimicrob Chemother.

[12] Zhang L, Fu Y, Xiong Z, Ma Y, Wei Y, Qu X (2018). Highly prevalent multidrug-resistant salmonella from chicken and pork meat at retail markets in Guangdong, China. Front Microbiol.

[13] Arbune M, Ciobotaru R, Voinescu D. Endovascular infection with Salmonella group C - a case report. Germs. https://www.ncbi.nlm.nih.gov/pmc/articles/PMC4570840/pdf/germs-05-03-099.pdf. 10.11599/germs.2015.1077 PMC4570840 26405678

[14] Sörelius Karl, di Summa Pietro G (2018). On the Diagnosis of Mycotic Aortic Aneurysms. Clinical Medicine Insights: Cardiology.

[15] Daye D, Walker TG (2018). Complications of endovascular aneurysm repair of the thoracic and abdominal aorta: evaluation and management. Cardiovasc Diagn Ther.

